# Educational interventions for physical activity among Brazilian adults: systematic review

**DOI:** 10.11606/s1518-8787.2021055003236

**Published:** 2021-12-01

**Authors:** Paulo Henrique Guerra, Hugo Falqueto Soares, Ana Beatriz Mafra, Izadora Czarnobai, Guilherme Airon Cruz, William Vinícius Weber, Juliano Cesar Huf Farias, Mathias Roberto Loch, Evelyn Helena Corgosinho Ribeiro

**Affiliations:** I Universidade Federal da Fronteira Sul Chapecó SC Brasil Universidade Federal da Fronteira Sul. Curso de Medicina. Chapecó, SC, Brasil; II Universidade de São Paulo Grupo de Estudos e Pesquisas Epidemiológicas em Atividade Física e Saúde da São Paulo SP Brasil Universidade de São Paulo. Grupo de Estudos e Pesquisas Epidemiológicas em Atividade Física e Saúde da São Paulo, SP, Brasil; III Universidade Federal da Fronteira Sul Programa de Pós-Graduação em Ciências Biomédicas Chapecó SC Brasil Universidade Federal da Fronteira Sul. Programa de Pós-Graduação em Ciências Biomédicas. Chapecó, SC, Brasil; IV Universidade Estadual de Londrina Programa de Pós-Graduação em Saúde Coletiva Londrina PR Brasil Universidade Estadual de Londrina. Programa de Pós-Graduação em Saúde Coletiva. Londrina, PR, Brasil

**Keywords:** Adult, Exercise, Health Education, Health Promotion, Systematic Review

## Abstract

**OBJECTIVE:**

To summarize the main evidence from educational interventions designed to increase levels of physical activity (PA) among Brazilian adults.

**METHODS:**

Systematic review of intervention studies carried out in Brazil that implemented educational components aimed at promoting increased levels of PA among adult populations (18 to 65 years old). In October 2020, systematic searches were conducted in six databases, and in the reference lists of the assessed studies.

**RESULTS:**

Of the initial 2,511 studies, nine were included in the synthesis. Samples with specific characteristics (such as social vulnerability, physical inactivity, and overweight or obesity) were observed, with a greater number of women. Five interventions (55.6%) occurred in primary healthcare settings (PHC) of the Brazilian Unified Health System (SUS). Only four studies (44.4%) described the pedagogical frameworks structuring the educational approaches, among which counseling was the most used strategy, such as those carried out through face-to-face meetings, home visits, lectures, and phone calls (n = 8; 88.9%). Positive results were observed in three different indicators: increase in weekly PA volume (n = 4); increase in leisure-time PA rate (n = 1); and increase in the proportion of women classified as “very active/active” (n = 1). Given the sampling specificities, the domain “participant selection” showed a high number of interventions with high risk of bias.

**CONCLUSIONS:**

Educational approaches engendered some positive effects on different PA indicators, notably counseling as the main strategy used and approaches involving other health themes, such as nutrition and stress. However, considering the several determinants of PA in Brazil, future interventions should be conducted in different locations of Brazil in order to evaluate, in a broader way, their implementation processes and articulation with the many professionals working in PHC.

## INTRODUCTION

Due to the multidimensional impacts it has on an individual’s life, physical activity (PA) has been identified as an important factor for human development^1^. More specifically, PA is recognized to be a determinant of several positive health indicators^2^, so that actions and policies for its promotion at the population level and throughout the life cycle have been advocated by several health agencies around the world^3,4^.

In the last decades, PA promotion has deserved much attention in the Brazilian public health agenda, especially for its introduction in the Brazilian Health System (in Portuguese: *Sistema Único de Saúde* - SUS) and in several national public policies^5^. However, despite this favorable institutional scenario, “promoting PA” is not a simple task in Brazil, since factors such as gender, income, education, and environment^6^ are determinants of its practice.

Primary healthcare (PHC) settings are, thus, potential settings for the implementation of strategies^9,10^ to reduce inequities in access to PA. In the SUS context is can potentially increase the completeness and resoluteness of healthcare, enhancing the ability to promote PA in contexts of different levels of social vulnerability^11,12^.

Literature also suggests that interventions based on educational processes^13^ entail favorable results to increase PA levels at different moments of life. This evidence, however, is mainly supported by data from interventions developed in high-income countries. Even with the existence of a systematic review of interventions in Latin American countries^14^, the relevance of a more specific synthesis of the Brazilian studies is justified, as it would enable a debate grounded in the Brazilian reality, besides pointing out possible advances toward future national surveys.

This study aims at summarizing the main evidence of educational interventions designed to increase the PA levels of Brazilian adults.

## METHODS

This study is characterized as a systematic literature review, with methodology and operational process based on “The Cochrane Handbook for Systematic Reviews of Interventions”^15^, and on the items of the “Preferred Reporting Items for Systematic Reviews and Meta-Analyses” (PRISMA)^16^ list, respectively. This review is part of a larger project named “Translation of evidence for decision making in the Brazilian public health system: a review of interventions aiming physical activity promotion”, registered in the PROSPERO database (CRD42015015993).

The inclusion criteria were designed based on the “PICOS” logic, considering that the synthesis would be made up of original scientific studies with the following characteristics: (I) Participants: adult populations, without disabilities, health conditions, or specific diseases, except for samples exclusively composed of participants with overweight or obesity; (II) Interventions: developed in Brazil, implemented in community settings (such as territories, PHC centers, outpatient clinics, and community organizations) and based on educational actions aimed to increase PA levels, regardless of the form of contact (face-to-face or remote meetings) and approach (individual or group); (III) Comparators: no restrictions were imposed regarding the activities performed by the control groups (if there were more than one control group, it was decided that the group that had received the least theoretical and/or practical content would be chosen); (IV) Outcome: PA levels, regardless of the PA indicators used (such as levels of moderate or vigorous PA, and number of steps a day), contexts observed (such as total PA, leisure time displacement time), and the instruments used to measure these (such as questionnaires and motion sensors); and (V) Study design: all types of intervention studies (“trials”), with no restrictions regarding the presences of randomization between groups and/or control group.

To retrieve the potential studies, we conducted: systematic searches in six electronic databases (Lilacs, Pubmed, Physical Education Index, Scielo, Scopus, and Web of Science) covering the available literature from the beginning until October 5, 2020, based on the strategy developed for Pubmed: (“physical activity”[Text Word] OR “exercise”[Text Word] OR “sport”[Text Word] OR “walk”[Text Word] OR “walking”[Text Word] OR “run”[Text Word] OR “running”[Text Word] OR “bike”[Text Word] OR “cyclying”[Text Word]) AND “Brazil”[Text Word] AND (“experimental study”[Text Word] OR “randomised controlled trial”[Text Word] OR “randomized controlled trial”[Text Word] OR “quasi-experimental”[Text Word] OR “clinical trial”[Text Word]). In addition to this strategy, manual searches were performed in the reference lists of the studies assessed by its full-texts.

The titles, abstracts and full texts assessments were performed independently by six researchers (AM, HF, GC, IC, JF and WW), supported by a senior researcher to clarify doubts, and establish consensus (PG). Data extraction was also conducted by the same six researchers, also independently, and supported by two senior researchers (ER and PG), on a spreadsheet initially divided into three domains: (I) descriptive features (such as intervention name/acronym, location, population description, and age/age group); (II) methods (such as recruitment and implementation location, group size at baseline and sample losses, description and strategies used in the intervention and control groups, intervention implementation team, and instruments and procedures used for measuring PA); and (III) PA-related outcomes (such as variables analyzed, procedures used for PA data analysis, and statistical magnitude and significance of findings based on “p-value”). Regarding the outcomes of interventions, effect sizes were considered for synthesis, as well as the results of the statistical comparison tests.

The risk of bias of the included studies was assessed by two researchers (ER and PG) using an adapted version of the EPHPP^17^ instrument, which assesses seven methodological domains of an intervention study: “selection bias”, “adjustment of confounding variables”, “methods used in data collection”, “losses and dropouts”, “intervention integrity”, “protocol used in the analysis”, and “use of intention to treat”.

## RESULTS

**Figura 1:**
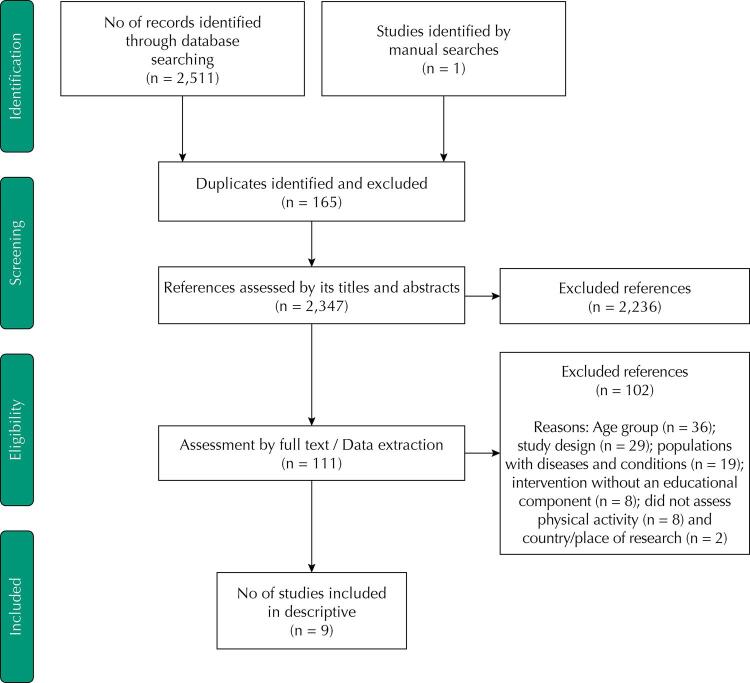
Fluxograma da revisão sistemática.

The electronic database searches resulted in 2,511 potentially relevant studies, of which 165 were initially identified as duplicates and thus excluded from the process (Figure 1). At the end of the evaluation by titles and abstracts, 110 studies were selected for reading of their full texts. Considering the exclusion of 102 of these, mainly due to “age group” (n = 36) and “study design” (n = 29), and the inclusion of one study retrieved by manual search on reference lists, the descriptive synthesis of the current review was composed from data of nine intervention studies conducted in Brazil^18^.

In Table 1, it can be observed that interventions were conducted in six Brazilian cities from five states, covering the Southeast (n = 6)^19,20,22,23,25,26^, South (n = 2)^18,24^ and Northeast (n = 1)^21^ regions, most of them conducted in cities of São Paulo state (n = 5–55.6%)^19,20,22,23,26^. Most of the interventions involved populations with mean age of 40 years (n = 6–66.7%)^18^, and women as the majority in all samples with available data (n = 8–88.9%)^18,19,21^.

**Table 1:** Descriptive features of the interventions included (n = 9).

Study	City-State (Data collection year)	Mean age (baseline)/%F	Intervention implementation site (Implementers)	Primary objective (Sample characteristics)
Assunção et al., 2010^18^	Pelotas-RS (2005–6)	40 (89%)	Nutrition Outpatient Care at School Hospital of UFPEL (Nutritionists)	Weight loss and control of risk factors for non-transmissible diseases (individuals with overweight or obesity)
Baba et al., 2017^19^	São Carlos-SP (2014–16)	48 (88%)	PHC-SUS (Professionals from health centers and Physical Education Professionals)	Physical activity (People living in regions of high vulnerability, and do not fulfill the PA recommendation)
Chaves; Oyama, 2007^20^	São Paulo-SP (nd)	40–59 (nd)	HCFMUSP Health promotion center (Research team)	Physical activity (Inactive people that participated in actions promoted by the outpatient service)
Costa et al., 2009^21^	Mutuípe-BA (2006–7)	≥ 35 (100%)	Community (Physical Education Professionals)	Weight loss (Participants of previous intervention)
Costa et al., 2015^22^	São Paulo-SP (2011–12)	18–39 (59%)	PHC-SUS Centers (Community Health Agents)	Physical activity (People served by the Family Health Strategy)
Ferreira et al., 2005^23^	São Caetano do Sul-SP (nd)	62 (100%)	City Hall Community Center (nd)	Physical activity (Participants of a regular exercises program)
Gomes; Duarte, 2008^24^	Florianópolis-SC (nd)	47 (83%)	PHC-SUS Centers (Research team and professionals of the Family Health Strategy)	Physical activity (People serviced by the Family Health Strategy)
Meurer et al., 2019^25^	Belo Horizonte-MG (2014–15)	62 (91%)	PHC-SUS Centers^a^ (Physical Education Professionals)	Physical activity, nutritional habits, and anthropometric measures (Health Academy Participants)
Ribeiro et al., 2017^26^	São Paulo-SP (nd)	18–39 (68%)	PHC-SUS Centers (Research team)	Physical activity (People in high socioeconomic vulnerability situation, and do not fulfill the PA recommendation)

^a^ Health Academy Programs Hubs; %F: percentage of women in the sample; PA: physical activity; PHC: primary healthcare; BA: Bahia; HCFMUSP: *Hospital das Clínicas* of the *Faculdade de Medicina da Universidade de São Paulo*; MG: Minas Gerais; nd: not described; RS: Rio Grande do Sul; SC: Santa Catarina; SP: São Paulo; SUS: Brazilian Unified Health System; UFPEL: *Universidade Federal de Pelotas*.

As for duration, interventions ranged from two^20^ to 12 months^21,26^, and five of them (55.6%) were developed over at least six months^18,19,21,22,26^ (Table 2). In two interventions, participants were followed up and evaluated for six months after the end of the intervention^19,26^. In regard of settings, we may highlight five interventions taking place in PHC settings of the SUS, either in health units^19,22,24,26^, or in centers of the Health Academy Program^25^, with great variability among people who delivered the interventions, as examples, professionals working in PHC-SUS settings, research teams, and specialists. Seven interventions had as their primary objective the increase in PA levels (77.8%)^19,20,22^.

**Table 2:** Synthesis of the elements part of the interventions (n = 9).

Randomized Controlled Trials
Assunção et al., 201018: Pedagogical framework: not described / Intervention (6 months): individual visits, with instructions for PA practice for 30 min/d, for at least 4 days a week, preferably aerobics and guidance for introduction of PA in the daily routine, involving the contexts of work, commuting and leisure. Nutrition content was also addressed. / Control: received individualized nutritional care
Meurer et al., 201925: Pedagogical framework: Social Cognitive Theory. / Intervention (4 months): weekly conversation wheels (60min) to discuss self-efficacy. Offer of educational material. Also worked with Nutrition themes. / Control: participated in the activities usually offered at the Health Academy Program center.

Non-Randomized Controlled Trials

Baba et al., 201719: Pedagogical framework: not described / Intervention (6 months, with evaluation of maintenance of results after 6 months of the end of the Intervention): five weekly sessions of health promotion, combining supervised PA practices (1h) and educational contents (discussions and readings after the practice and daily reports). Other health-related contents were also addressed / Control: did not receive information about PA recommendations.
Costa et al., 201522: Pedagogical framework: the teaching-learning process was guided by a social constructionist perspective. The ecological model for PA promotion at the community level, the transtheoretical model for behavior change applied to PA, and the National Policy of Continuing Education were also used. / Intervention (6 months): 29 Community Health Agents received training for PA promotion at the community level (12h), in order to support the guidance at home visits (90 people). / Control: did not receive any type of guidance/activity.
Ferreira et al., 200523: Pedagogical framework: not described / Intervention (3 months): a weekly guidance (5-10min) aimed at stimulating moderate PA, especially walking, for at least 30min/d, continuously or cumulatively, most days of the week. Educational material was offered. Also worked with Nutrition themes / Control: did not receive any kind of guidance/activity.
Gomes; Duarte, 200824: Pedagogical framework: Paulo Freire’s educational method. / Intervention (4 months): home visits with counseling and offer of educational material; 3 educational lectures (45-60min). / Control: did not receive any kind of guidance/activity.
Ribeiro et al., 201726: Pedagogical references: Social Cognitive Theory and Ecological Model for the promotion of PA. / Intervention (12 months, with evaluation of maintenance of results after 6 months of the end of the Intervention): 16 thematic-educational meetings on PA, healthy eating, and stress control (100min initial), with practices in the final 20 minutes. For the people who missed the meetings a phone call was made (20min) / Control: did not receive any kind of guidance/activity.

Non-controlled Trials

Chaves; Oyama, 200720: Pedagogical framework: not described / Intervention (2 months): receiving 5 phone calls, in the interval of 1 to 2 months, with counseling for PA, recording of information and discussions about difficulties for the practice and solutions found.
Costa et al., 2009^21^: Pedagogical framework: not described. / Intervention (12 months): three weekly sessions of supervised aerobic physical activity (1h each), with guidance to walk the rest of the days; bimonthly lectures (addressing food, nutrition, and health); broadcasting interviews on local radio (addressing healthy living habits). Nutrition content was also covered.

PA: physical activity; d: day; h: hour; min: minutes.

In four studies^22,24^ (44.4%), it was observed the description of pedagogical frameworks structuring the educational approaches, so that different approaches were combined in two studies^22,26^: the Social Cognitive Theory^25^ and Paulo Freire’s educational method^24^ (Table 2). Regarding the strategies adopted in the educational processes, counseling was used in eight interventions (88.9%), either through face-to-face meetings^18,19,21,23,26^, home visits^22,24^, lectures^24^, or phone calls^20^. Three interventions delivered educational materials^23^, while three interventions implemented hands-on activities^19,21,26^. In addition, five interventions addressing other health topics in their educational actions, such as nutrition^18,21,23,25,26^ and stress^26^ were also identified.

With the exception of the study by Meurer et al. (2019)^25^, all the interventions included used questionnaires to assess PA, with higher frequency of use of the IPAQ versions (n = 6)^18,19,21^. In addition to the questionnaire, two studies also used accelerometers to measure PA^19,26^. Samples ranged from 14^20^ to 291^25^ participants (Table 3).

**Table 3:** Synthesis of measurement instruments used and results related to increased levels of physical activity, by study design (n = 9).

Study	Physical activity measurement instrument	Number reviewed		Final result

		IG	CG	
Randomized Controlled Trials				
Assunção et al., 2010^18^	IPAQ-long version	120	121	Increase of 88 minutes per week in IG compared to CG (decrease of 39 minutes per week); p = 0.01.
Meurer et al., 2019^25^	Accelerometry (ActiGraph, GT3X and GT3X+)	135	156	Participants from IG showed a significant increase in moderate and vigorous PA compared to CG (effect size = 0.18). At the end of the Intervention, more IG participants met the PA recommendation compared with CG members.

Non-Randomized Controlled Trials				
Baba et al., 2017^19^	IPAQ and accelerometry	42	62	The IG showed an increase in PA levels (in leisure time, total and counts per minute), but without statistically significant differences in relation to the CG.
Costa et al., 2015^22^	IPAQ-long version (leisure and displacement modules)	79	79	From the comparison between IG and CG, no statistically significant differences were observed in the change of behavior change stages, PA levels in leisure time, and total PA (150 minutes per week).
Ferreira et al., 2005^23^	IPAQ	17^a^	15	The IG showed an increase in the weekly frequency of moderate PA practice in relation to the CG (Δ%: 49.6 versus -15.6; p < 0.05). No differences were observed between the groups regarding the duration (minutes per week) of activities, as well as in the variables walking frequency and duration.
Gomes; Duarte, 2008^24^	Baecke Questionnaire	44	43	Comparing the results between IG and CG, statistically significant changes (p < 0.05) were observed in the index of habitual PA at leisure.
Ribeiro et al., 2017^26^	IPAQ, Baecke questionnaire and accelerometry	54	49	We observed an increase in weekly minutes of PA during leisure time and in annual exercise, leisure time, and commuting scores. Six months after the end of the study, the group receiving the educational component showed an increase in the annual exercise score (0.2; 95% CI 0.1-0.4).

Non-Controlled Trials				
Costa et al., 2009^21^	IPAQ	69	–	An increase in the proportion of women in the “very active/active” category was observed at the end of follow-up (73.9%), with this difference being statistically significant when compared to the baseline value (30.4%) (p < 0.01).
Chaves; Oyama, 2007^20^	QDE	14	–	After all the counseling process, PA lasted 210 minutes or more in the week, and among the activities, the preferred one was walking.

PA: physical activity; CG: control group; IG: intervention group; IPAQ: International Physical Activity Questionnaire; QDE: questionnaire developed to the study.

^a^ Group that received guidance about physical activity and the nutritional component.

Regarding the effects, six interventions resulted in statistically significant data regarding PA in the following indicators: (I) increase in weekly PA volume^18,23,25,26^ - with highlights to the findings of Assunção et al. (2010)^18^, which showed an increase of 88 minutes a week in the intervention group compared to the control group (p = 0.01), of Meurer et al. (2019)^25^, which showed a significant increase of moderate and vigorous PA in the intervention group compared to the control group (Effect size = 0.18), and of Ribeiro et al. (2017)^26^, in which, six months after the end of the study, the group that received the educational component showed an increase in the annual exercise score (0.2; 95%CI 0.1-0.4); (II) increase in the index of habitual PA at leisure^24^; and, (III) increase in the proportion of women classified as “very active/active”, from 30.4% at baseline to 73.9% at the end of the intervention (Table 3), in Costa et al. (2009)^21^.

**Figura 2:**
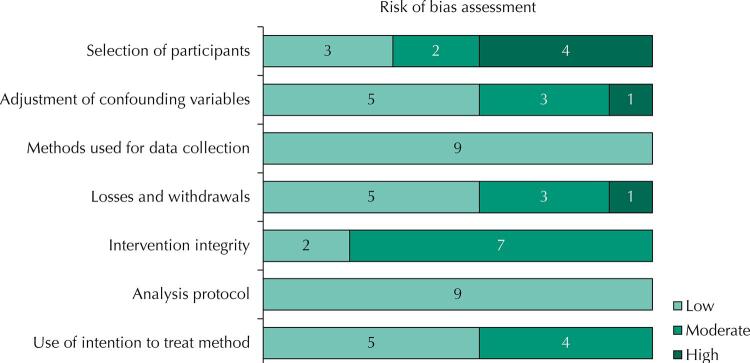
Análise do risco de viés das intervenções incluídas (n = 9).

According to Figure 2, methodological potentialities were observed in the domains “methods used in data collection” and “analysis protocol”, where all studies were classified as having “low” risk of bias. On the other hand, “participant selection” was the domain where a higher frequency of studies assessed as high risk were found, especially due to specificities in four samples (such as overweight/obese people^18^, people living in regions of high vulnerability^19,26^ and physically active^21,23,25^ or inactive^19,20,26^ people), which limit the generalizability of the original evidence. The domain “intervention integrity” showed seven studies rated as moderate risk of bias^20^, because they did not report the assessment of consistency of the intervention, and did not mention the risk of contamination between the groups – i.e., the influence caused by the possible proximity of people between the groups, so that those assigned to the control group may also be exposed to the actions conducted to the intervention group.

## DISCUSSION

Based on data from nine interventions conducted in six Brazilian cities, from three regions of the country, the current synthesis pointed out positive results in three different PA indicators: (I) increase in weekly PA volume^18,23,25,26^; (II) increase in leisure time PA index^24^; and (III) increase in the proportion of women ranked as “very active/active”^21^. Despite the great heterogeneity among pedagogical frameworks that supported the educational processes, counseling practice was the strategy most often adopted by the interventions, regardless of their format and the content approached, also highlighting approaches in other health-related topics, such as nutrition and stress.

Given the growth of academic production related to PA and health topic in Brazil^27^, the number of interventions can be considered to be low. However, this scarcity may be justified by the current context of cuts of budget for research in Brazil^28,29^, besides the fact that intervention studies demands longer time to be developed, as well as more funding and larger teams. Even so, regarding the results, what was observed in this synthesis supports the evidence of Heath et al. (2012)^13^ and Hoehner et al. (2013)^14^ regarding the effects of educational processes on interventions aimed at increasing the PA levels. The positive results observed in interventions conducted in PHC-SUS settings are also worth noticing^24,26^. International studies recommend interventions in PHC settings, considering the greater possibility of dissemination of a given strategy^9,10,30^.

Most studies did not report the pedagogical frameworks that supported the educational approaches. Considering that some of the strategies were conducted in the context of the PHC-SUS, which is grounded in premises of health promotion, this is an important gap, including for understanding their alignment with the main national policies, the paths of strategies, and the role of players involved throughout the implementation process. The four studies^22,24^ with available information based their interventions on different frameworks, either grouped or isolated, thus limiting more in-depth comparisons.

In view of the limitations perceived in the information-based approaches, it is suggested that future interventions also incorporate knowledge from Behavioral Economics^31^, particularly regarding its notion that knowledge, although important, is insufficient in most cases since it disregards the action of non-conscious processes that determine our choices^32^.

Furthermore, it is worth mentioning the importance of ecological or socio-ecological approaches and models, which include several factors from different “levels” that influence human behavior, in a perspective that goes beyond the understanding of health as a mere state of absence of disease^33^. Although educational strategies may play an important role in promoting PA levels, one should not lose sight of the fact that these should be implemented in parallel with the approach of many other factors^31^.

Regarding counseling, which was the most used educational strategy among the studies included, there are records on its positive influence on behavioral change stages^34^, as well as its effect on increasing PA levels^35^. A nationwide study indicates that PA counseling is a practice used by most physicians and nurses working in PHC^36^, even though many of them have little technical knowledge on PA-related issues.

As with the issue of pedagogical references, most of the studies included in the synthesis did not report important elements of the counseling processes, such as: concepts and topics approached, sequential logic subjects, as well as actions aimed at the broader concept of “PA promotion”, involving elements of identification and overcoming of barriers to the practice, for example. This finding reinforces the results found by Gagliardi et al. (2015)^37^ and, since the continuous offer of counseling is associated with maintaining high levels of PA in the long term, it is important to design strategies (such as training courses and guidelines) for the different professionals working in PHC^38^, in order to provide suitable theoretical and practical subsidies for strengthening it as a public health strategy^39^.

On the other hand, it should be emphasized that the practice of counseling - and any other strategy conducted alone - may be not enough to improve PA indicators: these themes need to be more present in people’s lives, either through information or other forms of interventions^33^. In other words, beyond the implementation of specific and/or isolated strategies, it is important that the “PA theme” be more present in people’s lives, since PA promotion is a cross-sector theme by nature.

In this spectrum, we highlight the importance of consistent planning of the built environment of cities, in order to expand people’s access to PA practice rooms. We could mention the opening of bike lanes which, besides physical demarcation of public roads, require traffic laws to ensure cyclists’ safety, as well as improvements in safety and lighting on public roads, favoring active transportation at different times of the day.

On the other hand, considering that most of the interventions included were developed in PHC-SUS settings, counseling for PA may be interconnected with the demands of other healthcare professionals, in the sense of more comprehensive guidance focused on healthy lifestyle habits, and the corresponding improvement of health conditions of a given person or group of people^39^. This suggestion is emphasized by this synthesis, since interventions were implemented by different specialists, not only by Physical Education professionals.

It is also worth mentioning the interventions that addressed other health themes, such as nutrition^18,21,23,25,26^ and stress^26^. Recognizing the emergence of the use of electronic appliances as an auxiliary tool to healthcare^40^, it can be suggested that future national studies test the introduction of applications with educational content and/or digital counseling under the prism of PHC. It is worth mentioning the promising results of interventions that use digital counseling for reducing systolic blood pressure^41^.

Even if it was not the objective of the study, but a gap perceived during the process of reading and extracting original data, it is recommended that future interventions report more deeply important internal and external elements such as adoption, scope, effectiveness, implementation and maintenance, as recommended by the RE-AIM instrument^42^. It is recognized that processes of consistent implementation, which articulate different health professionals with different levels of experience, allow greater generalization, and greater possibility of using this information in decision-making^42^. A previous systematic review suggests the weakness of reports on the Brazilian school-based interventions in the domains of adoption, implementation, and maintenance^43^.

Recognizing that the effectiveness of an intervention is directly related to how it is implemented, future studies are recommended to evaluate, in a broader perspective, the process of delivering these strategies, not disregarding the specificities of the SUS PHC settings. Thus, some starting points may be listed, such as: (I) prior recognition of the territories and their respective health needs, by approaching residents and community health agents; (II) permanent dialogue with the many PHC actors, in order to acknowledge the different possibilities of action, and encourage the articulated engagement of the multiprofessional team, from the initial proposal negotiations to the evaluation process; and, (III) expanded health approaches beyond the “AF theme”, developed in groups and complying with the logic of PHC.

This review has some limitations, such as (I) the little conceptual report of the elements that make up the intervention, (II) the congruence of strategies developed in SUS spaces with national policies on PHC, as well as the use of different (III) intervention designs, (IV) populations and contexts, and (V) PA indicators. Given these heterogeneities, we chose not to conduct the meta-analysis. On the other hand, as a major strength, it may be highlighted the more specific focus on educational strategies, which allowed the identification of important gaps, such as absence of reports on the pedagogical guidelines of the interventions, and on the consonance of protocols with the ideals of health promotion in Brazil.

Finally, the available set of Brazilian interventions suggests that the educational approaches produced some positive effects on different PA indicators, highlighting counseling as the main strategy used, and the approaches that involved other health themes, such as nutrition and stress. However, considering the several determinants of PA in Brazil, it is important that future interventions be conducted in different locations of the country, so as to comprehensively evaluate their processes of implementation and articulation with the different professionals working in PHC.
