# Supplementary material for: Educational interventions for physical activity among Brazilian adults: systematic review
Source: Rev Saude Publica. 2021 Dec 1;55:110. doi: 10.11606/s1518-8787.2021055003236 (PMC8664058; doi:10.11606/s1518-8787.2021055003236)
Supplement: Translation

# Translation: Intervenções educativas para atividade física em adultos brasileiros: revisão sistemática

**Authors:** Paulo Henrique Guerra, Hugo Falqueto Soares, Ana Beatriz Mafra, Izadora Czarnobai, Guilherme Airon Cruz, William Vinícius Weber, Juliano Cesar Huf Farias, Mathias Roberto Loch, Evelyn Helena Corgosinho Ribeiro

**Affiliations:** I Brasil Universidade Federal da Fronteira Sul. Curso de Medicina. Chapecó, SC, Brasil; II Brasil Universidade de São Paulo. Grupo de Estudos e Pesquisas Epidemiológicas em Atividade Física e Saúde da São Paulo, SP, Brasil; III Brasil Universidade Federal da Fronteira Sul. Programa de Pós-Graduação em Ciências Biomédicas. Chapecó, SC, Brasil; IV Brasil Universidade Estadual de Londrina. Programa de Pós-Graduação em Saúde Coletiva. Londrina, PR, Brasil

**Keywords:** Adulto, Exercício Físico, Educação em Saúde, Promoção da Saúde, Revisão Sistemática

## Abstract

**OBJETIVO:**

Sumarizar as principais evidências de intervenções educativas delineadas para o aumento dos níveis de atividade física (AF) em adultos brasileiros.

**MÉTODOS:**

Revisão sistemática de estudos de intervenção conduzidos no Brasil, que implementaram componentes educativos com a finalidade de promover o aumento dos níveis de AF em populações de adultos (18 a 65 anos). Em outubro de 2020, buscas sistemáticas foram conduzidas em seis bases de dados e nas listas de referências dos artigos avaliados.

**RESULTADOS:**

Dos 2.511 artigos iniciais, nove compuseram a síntese. Foram observadas amostras com características específicas (como vulnerabilidade social, inatividade física e sobrepeso ou obesidade), com maior número de mulheres. Cinco intervenções (55,6%) ocorreram nos cenários da atenção primária à saúde (APS) do Sistema Único de Saúde (SUS). Apenas em quatro estudos (44,4%) houve descrição dos referenciais pedagógicos estruturantes das abordagens educativas, dentro os quais o aconselhamento se configurou como a estratégia mais utilizada, como aquelas realizadas por meio de encontros presenciais, visitas domiciliares, palestras e chamadas telefônicas (n = 8; 88,9%). Resultados positivos foram observados em três distintos indicadores: aumento do volume semanal de AF (n = 4); aumento do índice de AF no lazer (n = 1); e aumento da proporção de mulheres classificadas como “muito ativas/ativas” (n = 1). Visto as especificidades amostrais, o domínio “seleção dos participantes” apresentou elevado número de intervenções com alto risco de viés.

**CONCLUSÕES:**

As abordagens educativas produziram alguns efeitos positivos em distintos indicadores de AF, destacando-se o aconselhamento como principal estratégia utilizada e as abordagens que envolveram outras temáticas de saúde, como nutrição e estresse. Contudo, frente aos diversos determinantes da AF no Brasil, é importante que futuras intervenções sejam conduzidas em variadas localizações do país, de forma que avaliem, de maneira mais ampla, seus processos de implementação e articulação com os distintos profissionais que atuam na APS.

## INTRODUÇÃO

Pelos impactos multidimensionais que ocasiona na vida de uma pessoa, a prática de atividade física (AF) tem sido apontada como um importante fator de desenvolvimento humano^1^. Mais especificamente, reconhece-se que a AF é determinante para diversos indicadores positivos de saúde^2^, de modo que ações e políticas para sua promoção, em nível populacional e ao longo do ciclo da vida, sejam preconizadas por diversas agências de saúde ao redor do mundo^3,4^.

Nas últimas décadas, a promoção de AF recebeu bastante atenção pela agenda da saúde pública brasileira, com destaques para sua introdução no Sistema Único de Saúde (SUS) e em várias políticas públicas nacionais^5^. Entretanto, à parte desse cenário institucional favorável, “promover AF” não é uma tarefa simples no país, visto que fatores como sexo, renda, escolaridade e ambiente^6^ são determinantes da sua prática.

Dessa forma, os cenários de atenção primária à saúde (APS) configuram-se como potenciais para a implementação de estratégias^9,10^, no sentido de reduzir as iniquidades no acesso às AF e, no âmbito do SUS, de ampliar a integralidade e a resolutividade do cuidado, potencializando a capacidade de promover a AF em contextos com distintos níveis de vulnerabilidade social^11,12^.

Complementarmente, a literatura sugere que intervenções fundamentadas em processos educativos^13^ produzem resultados favoráveis ao aumento dos níveis de AF, em diversos momentos da vida. Entretanto, essa evidência é amparada, quase predominantemente, por dados de intervenções conduzidas em países de alta renda. Mesmo com a existência de uma revisão sistemática de intervenções conduzidas nos países latino-americanos^14^, justifica-se a importância de uma síntese mais específica de estudos brasileiros, no sentido de oportunizar o debate de acordo com a realidade do país, assim como apontar possíveis avanços às futuras pesquisas nacionais.

Nesse sentido, este estudo objetiva sumarizar as principais evidências de intervenções educativas delineadas para o aumento dos níveis de AF de adultos brasileiros.

## MÉTODOS

Este estudo caracteriza-se como uma revisão sistemática da literatura, com metodologia e processo operacional baseados no *“The Cochrane Handbook for Systematic Reviews of Interventions”*^15^ e nos itens da lista *“Preferred Reporting Items for Systematic Reviews and Meta-Analyses”* (PRISMA)^16^, respectivamente. Esta revisão é parte de um projeto maior, intitulado “Tradução da evidência para a tomada de decisão no Sistema Único de Saúde: Revisão de intervenções com fins na promoção de atividade física” e registrado na base PROSPERO (CRD42015015993).

Os critérios de inclusão de estudos foram elaborados a partir da estratégia “PICOS”, considerando que a síntese seria composta por artigos científicos originais com as seguintes características: (I) Participantes: populações de adultos, sem deficiências, agravos à saúde ou doenças específicas, à exceção de amostras compostas apenas por participantes com sobrepeso ou obesidade; (II) Intervenções: desenvolvidas no Brasil, implementadas em ambientes comunitários (como territórios, centros de APS, ambulatórios e organizações comunitárias) e baseadas em ações educativas que objetivaram aumento dos níveis de AF, independentemente da forma de contato (por meio de encontros presenciais ou à distância) e da abordagem (individual ou grupal); (III) Comparadores: não foram impostas restrições em relação às atividades realizadas pelos grupos controles (na existência de mais de um grupo controle, estipulou-se que se escolheria o grupo que menos tivesse recebido conteúdos teóricos e/ou práticos); (IV) Desfecho: níveis de AF, independente dos indicadores de AF utilizados (como níveis de AF moderada ou vigorosa e número de passos por dia), contextos observados (como AF total, no tempo de lazer ou no deslocamento) e dos instrumentos utilizados para sua medida (como questionários e sensores de movimento); e (V) Delineamento de estudo: todos os tipos de estudos de intervenção (“ensaios”), sem restrições quanto às presenças de randomização entre grupos e/ou grupo controle.

Para a recuperação dos artigos potenciais, foram conduzidas: buscas sistemáticas em seis bases de dados eletrônicas (Lilacs, Pubmed, Physical Education Index, Scielo, Scopus e Web of Science), que abrangeram a literatura disponível desde seu início até 5 outubro de 2020, fundamentadas na estratégia desenvolvida para o Pubmed: (“physical activity”[Text Word] OR “exercise”[Text Word] OR “sport”[Text Word] OR “walk”[Text Word] OR “walking”[Text Word] OR “run”[Text Word] OR “running”[Text Word] OR “bike”[Text Word] OR “cyclying”[Text Word]) AND “Brazil”[Text Word] AND (“experimental study”[Text Word] OR “randomised controlled trial”[Text Word] OR “randomized controlled trial”[Text Word] OR “quasi-experimental”[Text Word] OR “clinical trial”[Text Word]). E, além dessa estratégia, foram realizadas buscas manuais nas listas de referências dos artigos avaliados na íntegra.

As avaliações de títulos e resumos e de textos integrais foram feitas por seis pesquisadores (AM, HF, GC, IC, JF e WW), de forma independente, com apoio de um pesquisador sênior para resolução de dúvidas e estabelecimento de consensos (PG). A extração dos dados também foi conduzida pelos mesmos seis pesquisadores, também de forma independente, e com apoio de dois pesquisadores sêniores (ER e PG), em uma planilha eletrônica inicialmente dividida em três domínios: (I) características descritivas (como nome/acrônimo da intervenção, local de realização, descrição da população e idade/faixa etária); (II) métodos (como local de recrutamento e implementação, tamanho dos grupos na linha de base e perdas amostrais, descrição e estratégias utilizadas no grupos de intervenção e controle, equipe de implementação da intervenção e instrumentos e procedimentos utilizados para mensuração da AF); e (III) resultados relacionados à AF (como variáveis analisadas, procedimentos utilizados na análise dos dados de AF e magnitude e significância estatísticas dos achados com base no “valor p”). Em relação aos resultados das intervenções, foram considerados para a síntese os tamanhos de efeito, assim como os resultados dos testes estatísticos de comparação.

O risco de viés dos artigos incluídos foi avaliado por dois pesquisadores (ER e PG) a partir do uso de uma versão adaptada do instrumento EPHPP^17^, que avalia sete domínios metodológicos de um estudo de intervenção: “viés de seleção”, “ajuste das variáveis de confusão”, “métodos utilizados na coleta de dados”, “perdas e desistências”, “integridade da intervenção”, “protocolo utilizado na análise” e “uso da intenção de tratar”.

## RESULTADOS

As buscas nas bases de dados eletrônicas resultaram em 2.511 artigos potencialmente relevantes, dos quais 165 foram inicialmente identificados como duplicatas e, dessa forma, excluídos do processo (Figura 1). Ao término da avaliação por títulos e resumos, 110 artigos remanesceram para a leitura de seus textos integrais. Considerando a exclusão de 102 destes, motivada principalmente pela “faixa etária” (n = 36) e pelo “delineamento de estudo” (n = 29), e a inclusão de um artigo recuperado por meio das buscas manuais nas listas de referências, a síntese descritiva da presente revisão foi composta a partir dos dados de nove estudos de intervenção conduzidos no Brasil^18^.

**Figura 1:** Fluxograma da revisão sistemática.

Na Tabela 1, pode-se observar que as intervenções foram realizadas em seis cidades brasileiras de cinco estados, que abrangem as regiões Sudeste (n = 6)^19,20,22,23,25,26^, Sul (n = 2)^18,24^ e Nordeste (n = 1)^21^, sendo a maioria delas conduzida em cidades do estado de São Paulo (n = 5; 55,6%)^19,20,22,23,26^. A maior parte das intervenções envolveu populações com idade média de40 anos (n = 6; 66,7%)^18^ e mulheres como maioria em todas as amostras com dados disponíveis (n = 8; 88,9%)^18,19,21^.

**Tabela 1:** Características descritivas das intervenções incluídas (n = 9).

Estudo	Cidade-Estado (Ano da coleta de dados)	Idade média (linha de base)/%F	Local de implementação da intervenção (Implementadores)	Objetivo primário (Característica da amostra)
Assunção et al., 2010^18^	Pelotas-RS (2005–6)	40 (89%)	Ambulatório de Nutrição do Hospital-Escola da UFPEL (Nutricionistas)	Redução de peso e controle dos fatores de risco para doenças não-transmissíveis (Pessoas com sobrepeso ou obesidade)
Baba et al., 2017^19^	São Carlos-SP (2014–16)	48 (88%)	Centros de APS-SUS (Profissionais dos centros de saúde e Profissionais de Educação Física)	Atividade física (Pessoas que vivem em regiões de alta vulnerabilidade e não atingem a recomendação de AF)
Chaves; Oyama, 2007^20^	São Paulo-SP (nd)	40–59 (nd)	Centro de promoção da saúde do HCFMUSP (Equipe de pesquisa)	Atividade física (Pessoas inativas que participavam de ações do serviço ambulatorial)
Costa et al., 2009^21^	Mutuípe-BA (2006–7)	≥ 35 (100%)	Comunidade (Profissionais de Educação Física)	Redução do peso (Participantes de intervenção prévia)
Costa et al., 2015^22^	São Paulo-SP (2011–12)	18–39 (59%)	Centros de APS-SUS (Agentes Comunitárias de Saúde)	Atividade física (Pessoas atendidas pela Estratégia Saúde da Família)
Ferreira et al., 2005^23^	São Caetano do Sul-SP (nd)	62 (100%)	Centro comunitário da prefeitura (nd)	Atividade física (Participantes de um programa regular de ginástica)
Gomes; Duarte, 2008^24^	Florianópolis-SC (nd)	47 (83%)	Centros de APS-SUS (Equipe de pesquisa e profissionais da Estratégia Saúde da Família)	Atividade física (Pessoas atendidas pela Estratégia Saúde da Família)
Meurer et al., 2019^25^	Belo Horizonte-MG (2014–15)	62 (91%)	Centros de APS-SUS^a^ (Profissionais de Educação Física)	Atividade física, hábitos nutricionais e medidas antropométricas (Participantes de Academias da Saúde)
Ribeiro et al., 2017^26^	São Paulo-SP (nd)	18–39 (68%)	Centros de APS-SUS (Equipe de pesquisa)	Atividade física (Pessoas em situação de alta vulnerabilidade socioeconômica e que não atingem a recomendação de AF)

^a^ Polos do Programa Academia da Saúde; %F: percentual de mulheres na amostra; AF: atividade física; APS: atenção primária à saúde; BA: Bahia; HCFMUSP: Hospital das Clínicas da Faculdade de Medicina da Universidade de São Paulo; MG: Minas Gerais; nd: não descrito; RS: Rio Grande do Sul; SC: Santa Catarina; SP: São Paulo; SUS: Sistema Único de Saúde; UFPEL: Universidade Federal de Pelotas.

Quanto à duração, as intervenções variaram entre dois^20^ e 12 meses^21,26^, e cinco delas (55,6%) foram desenvolvidas ao longo de pelo menos seis meses^18,19,21,22,26^ (Tabela 2). Em duas intervenções, os participantes foram acompanhados e avaliados por seis meses após o término da intervenção^19,26^. Quanto aos locais de pesquisa, podem ser destacadas cinco intervenções que ocorreram nos cenários de APS do SUS, seja em unidades de saúde^19,22,24,26^, seja em polos do Programa Academia da Saúde^25^, com grande variabilidade entre os implementadores, citando, como exemplos, profissionais que atuam nos cenários de APS-SUS, equipes de pesquisa e especialistas. Sete intervenções tiveram como objetivo primário o aumento dos níveis de AF (77,8%)^19,20,22^.

**Tabela 2:** Síntese dos elementos componentes das intervenções (n = 9).

Ensaio Controlado Randomizado
Assunção et al., 2010^18^: Referencial pedagógico: não descrito. / Intervenção (6 meses): consultas individuais, com orientações para a prática de AF por 30 min/d, por pelo menos 4 dias da semana, preferencialmente aeróbica e orientação para introdução de AF na rotina diária, envolvendo os contextos do trabalho, deslocamento e lazer. Também foram abordados conteúdos da Nutrição. / Controle: recebeu cuidado nutricional individualizado
Meurer et al., 2019^25^: Referencial pedagógico: Teoria Social Cognitiva. / Intervenção (4 meses): rodas de conversa semanais (60min) para discussão de autoeficácia. Oferta de material educativo. Também se trabalhou com temáticas da Nutrição. / Controle: participaram das atividades habitualmente ofertadas no polo do Programa Academia da Saúde.

Ensaios Controlados Não-Randomizados

Baba et al., 2017^19^: Referencial pedagógico: não descrito. / Intervenção (6 meses, com avaliação de manutenção dos resultados após 6 meses do término da intervenção): cinco sessões semanais de promoção de saúde, combinando práticas de AF supervisionadas (1h) e conteúdos educativos (debates e leituras após a prática e comunicados diários). Também foram abordados outros conteúdos relacionados à saúde. / Controle: não recebeu informações sobre as recomendações de AF.
Costa et al., 2015^22^: Referencial pedagógico: o processo ensino-aprendizagem foi orientado por uma perspectiva construcionista social. Também foram utilizados o modelo ecológico para a promoção da AF no nível da comunidade, o modelo transteórico para mudança de comportamento aplicado à AF e a Política Nacional de Educação Permanente. / Intervenção (6 meses): 29 Agentes Comunitárias de Saúde receberam formação para a promoção de AF no nível comunitário (12h), no sentido de apoiar a orientação nas visitas domiciliares (90 pessoas). / Controle: não recebeu nenhum tipo de orientação/atividade.
Ferreira et al., 2005^23^: Referencial pedagógico: não descrito. / Intervenção (3 meses): uma orientação semanal (5–10min) visando estimular AF moderadas, em especial a caminhada, por pelo menos 30min/d, de forma contínua ou acumulada, na maioria dos dias da semana. Oferta de material educativo. Também se trabalhou com temáticas da Nutrição. / Controle: não recebeu nenhum tipo de orientação/atividade.
Gomes; Duarte, 2008^24^: Referencial pedagógico: método educacional de Paulo Freire. / Intervenção (4 meses): visitas domiciliares com aconselhamento e oferta de material educativo; realização de 3 palestras educativas (45–60min). / Controle: não recebeu nenhum tipo de orientação/atividade.
Ribeiro et al., 2017^26^: Referenciais pedagógico: Teoria Social Cognitiva e Modelo Ecológico para a promoção da AF. / Intervenção (12 meses, com avaliação de manutenção dos resultados após 6 meses do término da intervenção): 16 encontros temático-educativos sobre AF, alimentação saudável e controle do estresse (100min iniciais), com práticas nos 20 minutos finais. Para as pessoas que faltaram nos encontros era realizada uma chamada telefônica (20min). / Controle: não recebeu nenhum tipo de orientação/atividade.

Ensaios Não-Controlados

Chaves; Oyama, 2007^20^: Referencial pedagógico: não descrito / Intervenção (2 meses): recebimento de 5 chamadas telefônicas, no intervalo de 1 a 2 meses, com aconselhamento para a AF, registro de informações e discussões sobre dificuldades para a prática e soluções encontradas.
Costa et al., 2009^21^: Referencial pedagógico: não descrito. / Intervenção (12 meses): três sessões semanais de atividades físicas aeróbicas supervisionadas (1h cada), com orientação para caminhadas no restante dos dias; palestras bimestrais (abordando alimentação, nutrição e saúde); transmissão de entrevistas na rádio local (abordando hábitos de vida saudáveis). Também foram abordados conteúdos da Nutrição.

AF: atividade física; d: dia; h: hora; min: minutos.

Em quatro estudos^22,24^ (44,4%), foi observada a descrição dos referenciais pedagógicos estruturantes das abordagens educativas, de forma que houve uso combinado de distintas abordagens em dois estudos^22,26^, da Teoria Social Cognitiva^25^ e do método educacional de Paulo Freire^24^Tabela 2. Sobre as estratégias adotadas nos processos educativos, a prática de aconselhamento foi utilizada em oito intervenções (88,9%), seja por meio de encontros presenciais^18,19,21,23,26^, de visitas domiciliares^22,24^, palestras^24^ ou de chamadas telefônicas^20^. Em três intervenções houve entrega de materiais educativos^23^ e em três intervenções também se observou a implementação de atividades práticas^19,21,26^. Complementarmente, foram identificadas cinco intervenções que abordaram, em suas ações educativas, outros tópicos de saúde, como nutrição^18,21,23,25,26^ e estresse^26^.

À exceção do estudo de Meurer et al. (2019)^25^, todas as intervenções incluídas utilizaram questionários para a avaliação da AF, percebendo-se maior frequência no uso das versões do IPAQ (n = 6)^18,19,21^. Em adição ao questionário, dois estudos também utilizaram acelerômetros para mensuração da AF^19,26^. As amostras variaram entre 14^20^ e 291^25^ participantes Tabela 3.

**Tabela 3:** Síntese dos instrumentos de medidas utilizados e resultados relacionados ao aumento dos níveis de atividade física, por delineamento de estudo (n = 9).

Estudo	Instrumento de mensuração da atividade física	Número analisado		Resultado final

		GI	GC	
Ensaios Controlados Randomizados				
Assunção et al., 2010^18^	IPAQ-versão longa	120	121	Aumento de 88 minutos por semana no GI em comparação ao GC (decréscimo de 39 minutos por semana); p = 0,01.
Meurer et al., 2019^25^	Acelerometria (ActiGraph, GT3X and GT3X+)	135	156	Participantes do GI apresentaram aumento significativo de AF moderadas e vigorosas em relação ao GC (Tamanho do efeito = 0,18). Ao final da intervenção, também se observou maior número de participantes do GI atendendo à recomendação de AF, em comparação com os integrantes do GC.

Ensaios Controlados Não-Randomizados				
Baba et al., 2017^19^	IPAQ e acelerometria	42	62	O GI apresentou aumento nos níveis de AF (no tempo de lazer, total e *counts* por minuto), mas sem diferenças estatisticamente significantes em relação ao GC.
Costa et al., 2015^22^	IPAQ-versão longa (módulos lazer e deslocamento)	79	79	Da comparação entre os GI e GC, não foram observadas diferenças estatisticamente significantes na alteração dos estágios de mudança de comportamento, nos níveis de AF no lazer e AF total (150 minutos por semana).
Ferreira et al., 2005^23^	IPAQ	17^a^	15	O GI apresentou aumento na frequência semanal de prática de AF moderadas em relação ao GC (Δ%: 49,6 versus -15,6; p < 0,05). Não foram observadas diferenças entre os grupos em relação à duração (minutos por semana) das atividades, assim como nas variáveis frequência e duração da prática de caminhada.
Gomes; Duarte, 2008^24^	Questionário de Baecke	44	43	Comparando os resultados entre os GI e GC, foram observadas mudanças estatisticamente significativas (p < 0,05) no índice de AF habitual no lazer.
Ribeiro et al., 2017^26^	IPAQ, questionário de Baecke e acelerometria	54	49	Observou-se aumento nos minutos semanais de AF no lazer e nos escores anuais de exercícios físicos, de lazer e de deslocamento. Após seis meses do término do estudo, o grupo que recebeu o componente educativo demonstrou aumento no escore anual de exercícios físicos (0,2; IC95% 0,1–0,4).

Ensaios Não-Controlados				
Costa et al., 2009^21^	IPAQ	69	–	Foi observado aumento da proporção de mulheres na categoria “muito ativas/ativas” ao final do seguimento (73,9%), sendo essa diferença estatisticamente significante quando comparada ao valor da linha de base (30,4%) (p < 0,01).
Chaves; Oyama, 2007^20^	QDE	14	–	Após todo o processo de aconselhamento, AF teve a duração igual ou superior a 210 minutos na semana e, dentre as atividades, a preferida foi a caminhada.

AF: atividade física; GC: grupo controle; GI: grupo intervenção; IPAQ: *International Physical Activity Questionnaire*; QDE: questionário desenvolvido para o estudo.

^a^ Grupo que recebeu orientações sobre atividade física e componente nutricional.

Em relação aos efeitos produzidos, seis intervenções resultaram em dados estatisticamente significantes em relação à AF, nos indicadores: (I) aumento do volume semanal de AF^18,23,25,26^ – com destaques para os achados de Assunção et al. (2010)^18^, que apresentou aumento de 88 minutos por semana no grupo intervenção em comparação ao grupo controle (p = 0,01), de Meurer et al. (2019)^25^, que apontou aumento significativo de AF moderadas e vigorosas no grupo intervenção em relação ao grupo controle (tamanho do efeito = 0,18) e de Ribeiro et al. (2017)^26^, em que, após seis meses do término do estudo, o grupo que recebeu o componente educativo demonstrou aumento no escore anual de exercícios físicos (0,2; IC95% 0,1–0,4); (II) aumento do índice de AF habitual no lazer^24^; e (III) aumento da proporção de mulheres classificadas como “muito ativas/ativas”, de 30,4% na linha de base para 73,9% ao término da intervenção (Tabela 3), em Costa et al. (2009)^21^.

Conforme a Figura 2, maiores potencialidades metodológicas foram observadas nos domínios “métodos utilizados na coleta de dados” e “protocolo de análise”, onde todos os artigos foram classificados tendo “baixo” risco de viés. Por outro lado, “seleção dos participantes” configurou-se como o domínio onde maiores fragilidades foram observadas, especialmente devido a especificidades em quatro amostras (como pessoas com sobrepeso/obesidade^18^, pessoas que vivem em regiões de alta vulnerabilidade^19,26^ e pessoas fisicamente ativas^21,23,25^ ou inativas^19,20,26^), que limitam a generalização da evidência original. O domínio “integridade da intervenção” foi classificado como moderado^20^ em sete estudos, por não relatarem a avaliação da consistência da intervenção e por não mencionarem o risco de contaminação entre os grupos, ou seja, a influência causada pela possível proximidade das pessoas entre os grupos, de modo que alocados no grupo controle também possam estar expostos às ações que são conduzidas ao grupo intervenção.

**Figura 2:** Análise do risco de viés das intervenções incluídas (n = 9).

## DISCUSSÃO

A partir dos dados de nove intervenções conduzidas em seis cidades brasileiras, de três regiões do país, a presente síntese apontou resultados positivos em três distintos indicadores de AF: (I) aumento do volume semanal de AF^18,23,25,26^; (II) aumento do índice de AF no lazer^24^; e (III) aumento da proporção de mulheres classificadas como “muito ativas/ativas”^21^. Mesmo com grande heterogeneidade entre os referenciais pedagógicos que alicerçaram os processos educativos, a prática do aconselhamento foi a estratégia mais adotada pelas intervenções, independentemente do seu formato e dos conteúdos abordados, destacando também abordagens em outras temáticas de saúde, como nutrição e estresse.

Visto o crescimento da produção acadêmica relacionada à área da AF e da saúde no país^27^, o número de intervenções pode ser considerado baixo. Contudo, essa escassez pode ser justificada frente ao contexto atual onde ocorrem cortes orçamentários relacionados à pesquisa no país^28,29^, além do fato de que o desenvolvimento de estudos de intervenção requer mais tempo, mais financiamento e maiores equipes. Mesmo assim, em relação aos resultados, o observado na presente síntese corrobora as evidências de Heath et al. (2012)^13^ e Hoehner et al. (2013)^14^ em relação aos efeitos de processos educativos em intervenções que visam ao aumento dos níveis de AF. Mais especificamente, também podem ser mencionados os resultados positivos observados nas intervenções conduzidas nos cenários de APS-SUS^24,26^. Estudos de porte internacional recomendam intervenções no contexto da APS, visto a maior possibilidade de disseminação de uma determinada estratégia^9,10,30^.

A maior parte dos estudos não relatou os referenciais pedagógicos que alicerçaram as abordagens educativas. Considerando que algumas das estratégias foram conduzidas no contexto da APS-SUS, que está alicerçada nas premissas da promoção da saúde, essa é uma lacuna importante, inclusive para a compreensão do seu alinhamento com as principais políticas nacionais, dos próprios rumos das estratégias e do papel dos atores envolvidos ao longo do processo de implementação. Os quatro estudos^22,24^ com informações disponíveis fundamentaram suas intervenções em distintos referenciais, seja de forma agrupada ou isolada, o que limita comparações mais aprofundadas.

Em vista das limitações percebidas nas abordagens baseadas em informação, sugere-se que futuras intervenções também incorporem saberes da Economia Comportamental^31^, em particular quanto a sua concepção de que o conhecimento, apesar de importante, é insuficiente na maioria dos casos, visto que não considera a ação dos processos não conscientes que determinam nossas escolhas^32^.

Além disso, vale mencionar a importância das abordagens e modelos ecológicos ou socioecológicos, que incluem diversos fatores, de diferentes “níveis”, que influenciam o comportamento humano, em uma perspectiva que caminha para além do entendimento de saúde como mero estado de ausência de doenças^33^. Dessa forma, mesmo que as estratégias educativas possam desempenhar um papel importante na promoção dos níveis de AF, não se pode perder de vista que elas devam ser implementadas em paralelo à abordagem de muitos outros fatores^31^.

Sobre a prática de aconselhamento, que se configurou como a estratégia educativa mais utilizada entre os estudos incluídos, há registro de sua influência positiva nos estágios de mudança de comportamento^34^, assim como de seu efeito no aumento dos níveis de AF^35^. Estudo de abrangência nacional aponta que o aconselhamento para a AF é uma prática utilizada pela maior parte dos médicos e enfermeiras que atuam na APS^36^, mesmo que boa parcela deles tenha pouco conhecimento técnico sobre os assuntos relacionados à AF.

Tal como na questão dos referenciais pedagógicos, a maior parte das pesquisas incluídas na síntese não relatou importantes elementos dos processos de aconselhamento, como: os conceitos e tópicos abordados, a lógica sequencial dos assuntos, assim como as ações voltadas ao conceito mais ampliado de “promoção da AF”, envolvendo elementos de identificação e superação de barreiras para a prática, por exemplo. Esse achado reforça os resultados de Gagliardi et al. (2015)^37^ e, visto que a oferta contínua de aconselhamento está associada à manutenção de elevados níveis de AF a longo prazo, é importante a formulação de estratégias (como cursos de formação e documentos orientadores) para os distintos profissionais que atuam na APS^38^, no sentido de oferecer os subsídios teóricos e práticos adequados para o seu fortalecimento enquanto estratégia de saúde pública^39^.

Por outro lado, cabe reforçar que a prática do aconselhamento – e de qualquer outra estratégia conduzida de forma isolada – pode não ser suficiente para a melhoria dos indicadores de AF: essas temáticas precisam estar mais presentes na vida das pessoas, seja por meio de informações ou mesmo de outras formas de intervenções^33^. Ou seja, para além da realização de estratégias mais pontuais e/ou isoladas, é importante que o “tema AF” esteja mais presente na vida das pessoas, visto que a promoção da AF é um objeto, por natureza, intersetorial.

Nesse espectro, destaca-se a importância do planejamento constante do ambiente construído das cidades, no sentido de ampliar o acesso das pessoas aos espaços de prática de AF. Como exemplo, pode-se mencionar a abertura de ciclofaixas, que, para além das demarcações físicas nas vias públicas, requerem leis de trânsito que garantam a segurança dos ciclistas, assim como melhorias na segurança e iluminação em vias públicas, favorecendo o transporte ativo nos distintos momentos do dia.

Por outro lado, considerando que a maior parte das intervenções incluídas ocorreu nos cenários de APS-SUS, sugere-se que o aconselhamento para a AF esteja interligado às demandas de outros profissionais de saúde, no sentido de uma orientação mais abrangente, voltada aos hábitos de vida saudáveis e respectiva melhora das condições de saúde de determinada pessoa ou de um grupo de pessoas^39^. Essa sugestão é acentuada pela presente síntese, uma vez que as intervenções foram implementadas por distintos especialistas, não apenas Profissionais de Educação Física.

Cabe destacar, também, as intervenções que abordaram outras temáticas de saúde, como nutrição^18,21,23,25,26^ e estresse^26^. Reconhecendo a emergência do uso de aplicativos eletrônicos como uma ferramenta auxiliar ao cuidado em saúde^40^, pode-se sugerir que futuros estudos nacionais testem a introdução de aplicativos, com conteúdos educativos e/ou aconselhamento digital sob o prisma da APS. Cabe mencionar os resultados promissores de intervenções que se utilizam de aconselhamento digital para a redução da pressão arterial sistólica^41^.

Mesmo que não tenha sido objetivo do estudo, mas uma lacuna percebida ao longo do processo de leitura e extração dos dados originais, recomenda-se que futuras intervenções tenham maior profundidade no relato de importantes elementos internos e externos, como adoção, alcance, efetividade, implementação e manutenção, tal como preconiza o instrumento RE-AIM^42^. Reconhece-se que processos de implementação consistentes, que articulem diferentes profissionais de saúde, com distintos níveis de experiência, permitem maior generalização e maior possibilidade do uso dessas informações na tomada de decisão^42^. Revisão sistemática prévia sugere a fragilidade dos relatos das intervenções brasileiras de base escolar nos domínios adoção, implementação e manutenção^43^.

Ao se reconhecer que a efetividade de uma intervenção está diretamente ligada à forma com que ela é implementada, recomenda-se que futuros estudos avaliem, em uma perspectiva mais abrangente, o processo de “entrega” dessas estratégias, sem desconsiderar as especificidades dos cenários de APS do SUS. Dessa forma, alguns pontos de partida podem ser elencados, como: (I) reconhecimento prévio dos territórios e suas respectivas necessidades de saúde, por meio de aproximações a moradores e agentes comunitárias de saúde; (II) diálogo permanente com os múltiplos atores da APS, no sentido de reconhecer as distintas possibilidades de ação e incentivar o envolvimento articulado da equipe multiprofissional, desde as tratativas iniciais da proposta até o processo de avaliação; e (III) abordagens ampliadas de saúde para além do “tema AF”, desenvolvidas em grupos e em respeito à lógica da APS.

Esta revisão possui algumas limitações, como (I) a pouca clareza conceitual dos elementos que compõem a intervenção, (II) a consonância das estratégias conduzidas nos espaços do SUS às políticas nacionais de APS, assim como a utilização de distintos (III) delineamentos de intervenções, (IV) populações e contextos e (V) indicadores de AF. Percebidas essas heterogeneidades, optou-se por não conduzir a metanálise. Por outro lado, como principal potência, pode ser destacado o olhar mais específico conferido às estratégias educativas, que permitiu a identificação de importantes lacunas, como a ausência de relatos sobre os norteadores pedagógicos das intervenções e sobre a consonância dos protocolos frente ao ideário de promoção da saúde no país.

Por fim, o conjunto disponível de intervenções brasileiras sugere que as abordagens educativas produziram alguns efeitos positivos em distintos indicadores de AF, destacando-se o aconselhamento como principal estratégia utilizada e as abordagens que envolveram outras temáticas de saúde, como nutrição e estresse. Contudo, frente aos diversos determinantes da AF no país, é importante que futuras intervenções sejam conduzidas em variadas localizações do país, de forma que avaliem, de maneira mais ampla, seus processos de implementação e articulação com os distintos profissionais que atuam na APS.
